# Data to reproduce and modify “An approach for screening single phase high-entropy alloys using an in-house thermodynamic database”

**DOI:** 10.1016/j.dib.2018.08.145

**Published:** 2018-08-30

**Authors:** Antonio João Seco Ferreira Tapia, Dami Yim, Hyoung Seop Kim, Byeong-Joo Lee

**Affiliations:** Department of Material Science and Engineering & Center for High-Entropy Alloys, Pohang University of Science and Technology (POSTECH), Pohang 37673, Republic of Korea

## Abstract

XRD raw data of the milled and heat-treated Co,Fe,Mn,Ni,Zn-containing high-entropy alloys (HEAs) is presented. Complete description of the quaternary, quinary and senary binary priority lists, including the names of the most relevant binary sets of elements for solid solution screening and the details of the parametric criterion used for making these lists are offered alongside a detailed thermodynamic description used to design Co,Fe,Mn,Ni,Zn-containing HEAs. In addition, a complete parametric study for quaternary, quinary and senary HEAs is shown. All the files and codes necessary to replicate and modify the previously mentioned results are available; commented by the authors in order to facilitate their usage. For further interpretation follow the research article: An approach for screening single phase high-entropy alloys using an in-house thermodynamic database (Tapia et al., 2018).

**Specifications Table**

**Table:** 

Subject area	*Materials Science*
More specific subject area	*High-entropy alloys screening*
Type of data	*Tables, XRD raw data, codes*
How data was acquired	*Parametric study, computational thermodynamics (Thermo-Calc), XRD and Excel VBA coding.*
Data format	*Raw and analyzed*
Experimental factors	*XRD analyses were conducted on mechanically alloyed samples*
Experimental features	*Phase identification of the milled powders and heat-treated samples was done using an X-ray diffractometer with Cu-Kα radiation.*
Data source location	*Pohang, Republic of Korea*
Data accessibility	*All data presented in this article*

**Value of the data**

• The data offers an insight into the recently developed concept of binary priority lists [1].
• A comprehensive parametric study for quaternary, quinary and senary HEAs is available for researchers interested in the design of HEAs.
• The given thermodynamic description for Co,Fe,Mn,Ni,Zn-containing HEAs can be used for exploring this alloy system compositional space, within and outside the face-centered cubic single phase solid solution stability range.
• Commented files and codes available make it possible to replicate or modify (in its entirety or individual steps, and in accordance to specific research goals) the simple yet effective screening methodology developed for single phase HEAs [1].

## 1 Data

In the Commented Files and Codes.xlsx file, the codes and data necessary to rapidly perform a thorough parametric study on multicomponent alloys as well as create the files necessary for the massive number of thermodynamic calculations performed for solid solution screening are available. A comprehensive parametric study for quaternary, quinary and senary HEAs is found in the Parametric Study.xlsx file. XRD raw data, thermodynamic descriptions of the Co,Fe,Mn,Ni,Zn-containing HEAs and details of the binary priority list concept are found in the Supplementary Data.xlsx file. A list of some of the FCC single phase high-entropy alloy systems found in the literature and predicted by our thermodynamic database are available in the FCC_predictions.docx file.

## 2 Experimental design, materials and methods

Excel VBA codes available in this dataset were employed to generate all the files necessary for the parametric study and solid solution screening. In order to fabricate the Co,Fe,Mn,Ni,Zn-containing HEAs, elemental powders of Co (>99.0% purity, 50 μm), Fe (>99.99% purity, 50 μm), Mn (>99.9% purity, 150 μm), Ni (>99.9% purity, 50 μm), and Zn (>99.9% purity, 50 μm) were mechanically alloyed in a planetary ball mill. Toluene was used as the process control agent to avoid agglomeration in the powders. The milling was carried out at 300 rpm for 30 h, using stainless balls of 5 mm diameter and a ball to powder weight ratio of 10:1. For sintering, the resultant ball-milled powders were compacted and sealed in a vacuum quartz tube to avoid oxidation. The compacted specimens were then heated in a furnace at 900 °C for 24 h. The heating rate of 50 °C/min was applied to increase the furnace temperature from room temperature to 900 °C. The sintered specimens were finally air cooled. The phase identification of the milled powders and heat-treated samples was done using an X-ray diffractometer with Cu-Kα radiation.
