# Supplementary material for: Data to reproduce and modify “An approach for screening single phase high-entropy alloys using an in-house thermodynamic database”
Source: Data Brief. 2018 Aug 30;20:1337–9. doi: 10.1016/j.dib.2018.08.145 (PMC6146562; doi:10.1016/j.dib.2018.08.145)
Supplement: Supplementary file 5 — Supplementary material [file mmc5.docx]

**Supplementary Information**

**An approach for screening single phase high-entropy alloys using an in-house thermodynamic database**

Antonio João Seco Ferreira Tapia, Dami Yim, Hyoung Seop Kim, Byeong-Joo Lee^∗^

Department of Material Science and Engineering & Center for High-Entropy Alloys

Pohang University of Science and Technology (POSTECH), Pohang 37673, Republic of Korea

^∗^ Corresponding author: E-mail: [calphad@postech.ac.kr](mailto:calphad@postech.ac.kr)

Table S1. List of some of the FCC single phase high-entropy alloy systems found in the literature predicted by our screening methodology and in-house thermodynamic database.

| **Alloy composition** | $\boldsymbol{\delta}$ | ${\boldsymbol{\Delta}\boldsymbol{H}}_{\boldsymbol{mix}}$ **(kJ/mol)** | **Reference** |
| --- | --- | --- | --- |
| CoCrCu_0.5_FeNi | 1.0618 | 0.4938 | [1] |
| CoCrCuFeNi | 1.0715 | 3.2000 | [2] |
| Al_0.2_CrCuFeNi_2_ | 2.6903 | 0.1183 | [3] |
| CoCrFeMnNi | 0.9241 | -4.1600 | [4] |
| Al_0.3_CoCrCuFeNi | 3.1462 | 0.1566 | [2] |
| Al_0.5_CoCrCuFeNi | 3.8167 | -1.5207 | [2] |
| Al_0.3_CoCrFeNi | 3.4903 | -7.2688 | [5] |
| Al_0.5_CrCuFeNi_2_ | 3.8167 | -2.5124 | [6] |
| CoCuFeNiV | 2.8811 | -2.2400 | [7] |
| CoCrFeNi | 1.0289 | −3.7500 | [8] |
| CoFeMnNi | 0.6594 | −4.0000 | [8] |
| CoCrMnNi | 0.9720 | −5.5000 | [8] |
| Al_0.25_CoCrFeNi | 3.2521 | -6.7543 | [5] |
| Al_0.3_CoCrFeNi | 3.4903 | -7.2688 | [9] |
| Al_0.375_CoCrFeNi | 3.7990 | -7.9935 | [5] |
| Al_0.5_CoCrCu_0.5_Fe_2_Ni | 3.6731 | -3.5278 | [3] |
| Al_0.5_CoCrCu_0.5_Fe_3_Ni | 3.4134 | -2.8367 | [3] |
| Al_0.5_CoCrCu_0.5_Fe_3.5_Ni | 3.3026 | -2.5778 | [3] |
| Al_0.2_CrCuFeNi_2_ | 2.6903 | 0.1183 | [10] |
| Al_0.4_CrCuFeNi_2_ | 3.5121 | -1.7010 | [10] |
| Al_0.5_CrCuFeNi_2_ | 3.8167 | -2.5124 | [10] |
| Al_0.25_CoCrCu_0.75_FeNi | 3.0032 | -0.7100 | [3] |
| Al_0.5_CoCrCu_0.5_FeNi | 4.0026 | -4.6000 | [3] |
| CoCrFeMo_0.3_Ni | 2.9245 | -4.1536 | [9] |
| Al_0.3_CoCrFeMo_0.1_Ni | 3.7427 | -7.2624 | [9] |
| CoCuFeMnNi | 0.9241 | 1.7600 | [11] |
| CoCuFeNi | 1.0289 | 5.0000 | [12] |
| Al_0.5_CoCrCuFeNiV_0.2_ | 4.0625 | -2.4992 | [3] |

**References**

[1] C.C. Tung, J.W. Yeh, T. tsung Shun, S.K. Chen, Y.S. Huang, H.C. Chen, On the elemental effect of AlCoCrCuFeNi high-entropy alloy system, Mater. Lett. 61 (2007) 1–5. doi:10.1016/j.matlet.2006.03.140.

[2] C.-J. Tong, Y.-L. Chen, J.-W. Yeh, S.-J. Lin, S.-K. Chen, T.-T. Shun, C.-H. Tsau, S.-Y. Chang, Microstructure characterization of Al x CoCrCuFeNi high-entropy alloy system with multiprincipal elements, Metall. Mater. Trans. A. 36 (2005) 881–893. doi:10.1007/s11661-005-0283-0.

[3] Y.F. Ye, Q. Wang, J. Lu, C.T. Liu, Y. Yang, High-entropy alloy: challenges and prospects, Mater. Today. 19 (2016) 349–362. doi:10.1016/j.mattod.2015.11.026.

[4] B. Cantor, I.T.H. Chang, P. Knight, A.J.B. Vincent, Microstructural development in equiatomic multicomponent alloys, Mater. Sci. Eng. A. 375–377 (2004) 213–218. doi:10.1016/j.msea.2003.10.257.

[5] Y.F. Kao, T.J. Chen, S.K. Chen, J.W. Yeh, Microstructure and mechanical property of as-cast, -homogenized, and -deformed AlxCoCrFeNi (0 ≤ x ≤ 2) high-entropy alloys, J. Alloys Compd. 488 (2009) 57–64. doi:10.1016/j.jallcom.2009.08.090.

[6] C. Ng, S. Guo, J. Luan, Q. Wang, J. Lu, S. Shi, C.T. Liu, Phase stability and tensile properties of Co-free Al0.5CrCuFeNi2high-entropy alloys, J. Alloys Compd. 584 (2014) 530–537. doi:10.1016/j.jallcom.2013.09.105.

[7] Y. Zhang, Y. neng Lv, On the nonisospectral modified Kadomtsev-Peviashvili equation, J. Math. Anal. Appl. 342 (2008) 534–541. doi:10.1016/j.jmaa.2007.12.032.

[8] Z. Wu, H. Bei, F. Otto, G.M. Pharr, E.P. George, Recovery, recrystallization, grain growth and phase stability of a family of FCC-structured multi-component equiatomic solid solution alloys, Intermetallics. 46 (2014) 131–140. doi:10.1016/j.intermet.2013.10.024.

[9] T.T. Shun, C.H. Hung, C.F. Lee, Formation of ordered/disordered nanoparticles in FCC high entropy alloys, J. Alloys Compd. 493 (2010) 105–109. doi:10.1016/j.jallcom.2009.12.071.

[10] S. Guo, C. Ng, C.T. Liu, Anomalous solidification microstructures in Co-free Al xCrCuFeNi2 high-entropy alloys, J. Alloys Compd. 557 (2013) 77–81. doi:10.1016/j.jallcom.2013.01.007.

[11] L. Liu, J.B. Zhu, L. Li, J.C. Li, Q. Jiang, Microstructure and tensile properties of FeMnNiCuCoSnxhigh entropy alloys, Mater. Des. 44 (2013) 223–227. doi:10.1016/j.matdes.2012.08.019.

[12] L. Liu, J.B. Zhu, C. Zhang, J.C. Li, Q. Jiang, Microstructure and the properties of FeCoCuNiSnxhigh entropy alloys, Mater. Sci. Eng. A. 548 (2012) 64–68. doi:10.1016/j.msea.2012.03.080.
